# About the Editor of the Cosmos, Etc.

**Published:** 1866-02

**Authors:** Geo. Watt


### Correspondence.

---

### ABOUT THE EDITOR OF THE "COSMOS," ETC.

ED. REGISTER:—On the 23d of October last, I sent an article to the "Dental Cosmos." So long was I in hearing of it, that I took for granted that it had gone *under* the editor's table; and perhaps it did, and was raked out by a higher power. Such has been the experience of much finer articles than it, as, for example, the beautiful song "Rain on the Roof." The January number, however, produced the article; and then the delay was explained: It was not regarded as sufficiently *truthful* to appear alone; and the antidote, in the shape of half a page of editorial damnation, was not ready to accompany it. This article was entitled "PHYSIOLOGY AMONG DENTISTS;" and its sole object was to stir up our profession to a more earnest and more extended cultivation of physiological science. To accomplish this, it was thought necessary to demonstrate the fact that it had been, to some extent, neglected. The Chicago meeting of the AMERICAN DENTAL ASSOCIATION was referred to as follows: "A prominent and talented member told us of queen bees being reared from the larvæ of drones, simply by a change of diet. * * * A physiologist can appreciate the fact, that an improved diet may develop genital organs that would lie dormant under a less generous regimen, but that the *sex* can be changed by a variation of food, is new to him."

To this the editor of the "Cosmos" responds as follows: "Had such an assertion been made, the proper place to have corrected it would have been on the spot; but no such position was assumed by any one at the meeting."

Here is contradiction as direct and unequivocal as the familiar "Katy-did" and "Katy-didn't." I wrote to the editor that the question of veracity between us was rather too sharp to rest on, and requested a retraction or explanation. (A copy of my letter is not before me). He responds: "I do not regard the question between us as one of '*veracity*,' but of *apprehension*," which would be quite satisfactory if the history of the affair, and the general character of the editorial, would permit such statement to be readily believed. Look at the facts: The profession was once somewhat acquainted with me as a writer. That acquaintance had to be broken off. Too prolonged over-taxation of the brain rendered relaxation necessary. Seven years of hard labor, daily, and mostly long into the night, with but three days of mental relaxation during the time, proved too much; and rest was required. Passing through, (no, only partly through), a severely afflictive ordeal, the brain was restored to its normal condition; and, with gratitude and thankfulness, I again took up the pen. I wrote an article for the "Cosmos"—the first I ever wrote for it. And I believe there were at least ten, and possibly twenty, *readers* of the "Cosmos," who were glad to know that I was again able to write; and I presume, as well as I can judge from manifestations, that the "Cosmos" has not more than *one writer*, who is sorry that I can wield the pen once more. And, now, readers of the "Register," especially those of you who have read the "Cosmos," too, did you, in all the history of professional journalism, ever know any writer, old or young, experienced or inexperienced, get such an editorial welcome? The editor had the article more that two months before its publication. If he could not insert it without the statement, virtually made, that one of its assertions is false, and the rest contrary to fact, why did he not reject it—throw it under the table, burn or return it? It was received by him in October. The December number appears. No notice that it is not all right." "Proof" is sent for correction,—no intimatiom that any statement needs modification. But, after two months of editorio-gestation, he is safely accouched of a "full-grown offspring," which, true to the instincts of its maternity, thus *welcomes* an old writer to the parental mansion, on his first visit: "Had such an assertion been made, the proper place to have corrected it would have been on the spot; but no such position was assumed by any one at the meeting" And "The sweeping objections to the general character of the discussion on Physiology in the Association are equally open to exceptions; but it is not necessary to dwell upon them, as the facts to the contrary * * * are so palpable and easy of recogniton."

Between these two charges, he gives a little statement about the raising of queen bees, which is as correct and clear as would be expected from a boy or girl in the intermediate department of our public schools, and not far behind a lecture I once heard on the eye, from the same renowned teacher, during a visit to our north-western metropolis. In his private letter, he says he is very familiar with the bee subject; and we found him so with the other subject; for he was always able to *read* the order of colors, as presented by the prismatic decomposition of light, but in no instance, during that hour, able to *state* it without close adherence to his notes.

A pious man, but a bad reader, was sorely puzzled, in his family devotions, with the names, "Shadrach, Meshach, and Abednego." When they were cast into the furnace, he expressed a hope that he was done with them. But reading farther, he manifested his surprise and disappointment in a shrill whistle, adding, "Here come the hard-named boys out to torment me again!" The *cordiality* with which the editor of the "Cosmos" welcomes me, from the *furnace* of affliction, back to the list of dental writers, is very similar. I infer, either that he regrets my partial recovery, or that somebody has hit him with a sour apple.

But if he regards the question between us as only one of "apprehension," could he not have admitted the possibility of himself being mistaken? Yet he does not do it, but charges me with false positions throughout my article. Does he not know that he did not hear all that was said at the Chicago meeting? Does he not know that a man may sit beside a clock, more than an hour, and not hear it strike? A man who had to speak oftener than any other three men at a meeting, who had to give school-boy lectures, who had to pass a rehash of old editorials for an original paper, who felt impelled to make himself so miscellaneously officious and useful, that the common inquiry was "When is that man going to hold another meeting of the Association?" a man so overwhelmed might fail to hear a word or two. He might even fail to notice that a speaker said "drone," instead of *worker*, when all else was correct in the speech. That even the infallible editor of the "Cosmos" might fail to hear every thing, may be illustrated thus: Another member and myself had a dispute as to whether a young crab, or crawfish, would continue to grow, while a lost arm was being replaced, by the growth of a new one. The production of a new member, to replace the lost one, was assumed as a thing known to every body. We both talked about it; and, near the close of the special debate on that subject, the editor of the "Cosmos" kindly furnished me a card, by way of assistance, on which he had written in substance, "Don't forget the *important* fact that the *crustacea* have the power of reproducing lost members." And he heard all the debate? He was sitting close by.

As to a failure to correct the mistake "on the spot," I have simply to say that I had already spoken *twice* on the subject; (as often as allowed by rule, I believe) and I thought there was "a more excellent way." I privately pointed out, to the speaker, his inadvertence, that he might correct it himself; but I suppose he forgot to do it. He admitted the mistake, and thanked me for correcting it as I did. That the editor failed to hear it, is no proof that it was not uttered. Two witnesses swore that they saw Tim strike Mike, and showed the stick with which he struck him. A dozen testified that they didn't see Tim strike Mike, and each showed a stick with which he didn't strike him. But, by some mishap, Tim was convicted.

This may look like giving too much attention to a *small affair*. But there was once another "*Little Mc.*"—a very little man, smaller, perhaps, than the editor of the "Cosmos;" and, though naturally calling for no special attention, yet by virtue of an artificial position, accidently obtained, he was able, for a long time, to paralyze the energies of a great nation. Our *little* Mc., by virtue of his *artificial*, or accidental position, at the head of a leading journal, may do much mischief to our profession, and hence is entitled to attention that would not otherwise be warranted.

The readers of the "Register" *are my friends.* If I am set right with them, I have gained my main point. The readers of the "Cosmos" ought to feel grateful to me. If I had not written that article, their January number would have had no "EDITORIAL."

Yours Truly,

GEO. WATT.

